# Distribution of alpha1 antitrypsin rare alleles in six countries: Results from the Progenika diagnostic network

**DOI:** 10.1186/s40246-023-00497-1

**Published:** 2023-06-05

**Authors:** José Luis Lopez-Campos, Noelia Rapun, Karen Czischke, José R. Jardim, Mariano Fernandez Acquier, Abraham Ali Munive, Hakan Günen, Estrella Drobnic, Marc Miravitlles, Lourdes Osaba

**Affiliations:** 1 Unidad Médico-Quirúrgica de Enfermedades Respiratorias, Instituto de Biomedicina de Sevilla (IBiS), Hospital Universitario Virgen del Rocío/Universidad de Sevilla, Avda. Manuel Siurot, S/N, 41013 Seville, Spain; 2 Progenika Biopharma, a Grifols Company. Derio, Vizcaya, Spain; 3 Departamento de Neumología, Clínica Alemana de Santiago, Universidad del Desarrollo, Santiago, Chile; 4 Centro de Reabilitação Pulmonar da Escola Paulista de Medicina da Universidade Federal de São Paulo (EPM/Unifesp), São Paulo, Brazil; 5 Servicio de Neumonología, Hospital Cetrángolo, Vicente López, Buenos Aires, Argentina; 6 Departamento Médico, Fundación Neumológica Colombiana, Bogotá, D.C. Colombia; 7 University of Health Sciences, Süreyyapaşa Research and Training Center for Chest Diseases and Thoracic Surgery, Istanbul, Turkey; 8 Scientific & Medical Affairs, Grifols, Barcelona, Spain; 9 Servicio de Neumología, Hospital Universitari Vall d’Hebron/Vall d’Hebron Research Institute (VHIR), Vall d’Hebron Barcelona Hospital Campus, Barcelona, Spain; 10 CIBER de Enfermedades Respiratorias (CIBERES), Instituto de Salud Carlos III, Madrid, Spain

**Keywords:** Alpha1 antitrypsin deficiency, Diagnosis, Rare alleles, Null alleles, Frequency

## Abstract

**Background:**

Knowledge of the frequency of rare *SERPINA1* mutations could help in the management of alpha1 antitrypsin deficiency (AATD). The present study aims to assess the frequencies of rare and null alleles and their respiratory and hepatic pathogenicity.

**Methods:**

This is a secondary analysis of a study that evaluated the viability of the Progenika diagnostic genotyping system in six different countries by analyzing 30,827 samples from cases of suspected AATD. Allele-specific genotyping was carried out with the Progenika A1AT Genotyping Test which analyses 14 mutations in buccal swabs or dried blood spots samples. *SERPINA1* gene sequencing was performed for serum AAT-genotype discrepancies or by request of the clinician. Only cases with rare mutations were included in this analysis.

**Results:**

There were 818 cases (2.6%) carrying a rare allele, excluding newly identified mutations. All were heterozygous except for 20 that were homozygous. The most frequent alleles were the M-like alleles, PI*M_malton_ and PI*M_heerlen_. Of the 14 mutations included in the Progenika panel, there were no cases detected of PI*S_iiyama_, PI*Q0_granite falls_ and PI*Q0_west_. Other alleles not included in the 14-mutation panel and identified by gene sequencing included PI*M_würzburg_, PI*Z_bristol_, and PI*Z_wrexham_, and the null alleles PI*Q0_porto_, PI*Q0_madrid_, PI*Q0_brescia_, and PI*Q0_kayseri_.

**Conclusions:**

The Progenika diagnostic network has allowed the identification of several rare alleles, some unexpected and not included in the initial diagnostic panel. This establishes a new perspective on the distribution of these alleles in different countries. These findings may help prioritize allele selection for routine testing and highlights the need for further research into their pathogenetic role.

## Introduction

In recent decades, the number of described mutations of the *SERPINA1* gene associated with alpha1 antitrypsin deficiency (AATD) has increased considerably. Beyond the two most frequent mutations, the S mutation (c.863A > T) and the Z mutation (c.1096G > A) [1], the number of described variants has risen to more than 500 [2]. Usually, the identification of these rare mutations is initiated by a discrepancy between the serum AAT level and the mutation found by direct genotyping [3]. For this reason, rare mutations are usually considered to be pathogenetic. However, most have been described in individual cases without thorough examination of their pathogenetic capacity [4]. Moreover, the frequency of these rare mutations in a large population of AATD patients has not been consistently described. It is clear that the mutation should be identified in AATD cases with significant pulmonary or hepatic involvement [5]. Knowing the frequency of these rare mutations could help in the management of the disease and in prioritizing allele identification in routine practice. This could also highlight the gaps in our understanding of the pathophysiologic behavior of these mutations.

Recently, our group published the results of a new system for AATD diagnosis based on buccal swabs and dried blood spots samples. After analyzing more than 30,000 samples from six countries, the study showed this diagnostic procedure was feasible and suitable for the genetic diagnosis of AATD [1, 3]. The implementation of this AATD diagnostic network has revealed that there are 14 mutations that can explain the majority of the pathological cases of this disease. Using the data from this study, the present analysis describes the frequencies of rare alleles and relate them to the available data on their respiratory and hepatic pathogenicity. These results will help understand the epidemiological importance of the mutations in each geographic area and will highlight the research needed for a more complete understanding of the pathogenetic potential of these mutations.

## Methods

This is a secondary analysis of the data from a study evaluating the Progenika diagnostic system (Progenika Biopharma, Derio, Vizcaya, Spain) in 30,827 samples from patients with suspected AATD from six different countries. This diagnostic network found mutations in 9,528 (30.9%) of the samples. The methodology has been previously described [1]. Briefly, this was an observational, cross-sectional analysis analyzing the anonymized data included on the Progenika web platform (https://grifolsalpha1test.com/) from March 12, 2018, to January 10, 2022. The collection kits for sampling with the dried blood spots or buccal swabs were provided to participating centers free of charge by Grifols (Barcelona, Spain) upon request from the treating physicians. For the current analysis, samples from Argentina, Brazil, Chile, Colombia, Spain, and Turkey were analyzed. The samples were registered on the web platform through a unique code associated with each sample collection kit and sent by post to the reference laboratory at the Progenika headquarters.

When registering the sample on the website, clinicians were asked to include some clinical data about the patient including age, smoking status (smoker, former smoker or never smoker), serum AAT level, and forced expiratory volume in one second (FEV_1_, expressed as a percentage of its predicted value), and the reasons for requesting the test. Although inclusion of these data was not mandatory, the AAT level was considered for concordance with the genotype and, if not concordant, the *SERPINA1* gene was sequenced. Per the Spanish guidelines [6], AAT levels ≤ 50 mg/dl were considered a severe deficiency.

Allele-specific genotyping was carried out with the Progenika A1AT Genotyping Test. The test uses polymerase chain reaction amplification to obtain large amounts of target sequences from the *SERPINA1* gene. The Luminex® 200 system to detect previously labeled amplified fragments, as previously described [3]. The test and OCR100 buccal swabs used to collect the samples are CE marked (European Conformity) and United States Food and Drug Administration approved. The test is intended for use with genomic DNA extracted from human whole blood samples collected as dried blood spots or from human buccal swab samples using ORAcollect Dx OCD-100.

The test can identify the 14 most frequent deficiency variants of the *SERPINA1* gene, namely PI*S, PI*Z, PI*I, PI*M_procida_, PI*M_malton_, PI*S_iiyama_, PI*Q0_granite falls_, PI*Q0_west_, PI*Q0_bellingham_, PI*F, PI*P_lowell_, PI*Q0_mattawa_, PI*Q0_clayton_, and PI*M_heerlen_. When none of the 14 alleles was found, the result was noted as negative and interpreted as an M allele. The absence of any of these 14 alleles suggests with over 99% probability that the genotype corresponds to PI*M, unless there was a discrepancy with AAT levels. In those cases, gene sequencing was conducted.

For the current analysis, only cases with rare mutations identified by the Progenika diagnostic system were included. Accordingly, those cases with genotypes exclusively resulting from a combination of S or Z alleles (MS, MZ, SS, SZ and ZZ) were excluded from this analysis. Newly identified mutations not previously described were also excluded. After the identification of all rare alleles, we performed a non-systematic review of the literature looking for information on these rare alleles by searching for the name of the allele in PubMed.

## Results

The number of patients with rare variants was 818 (2.7% out of 30,827 samples; 8.6% out of 9,528 carrying any mutation). The flowchart of the distribution of the samples is available from a previous analysis [1]. The number of patients carrying rare alleles is listed by country in Table 1. Severe AAT deficiency was seen in 572 patients (9.8% of those with serum AAT values). All cases were heterozygous except for the following (*n* = 20): 1 homozygous PI*M_procida_ (*n* = 1), homozygous PI*M_malton_ (*n* = 13), homozygous PI*M_heerlen_ (*n* = 1), homozygous PI*P_lowell_ (*n* = 4), and homozygous PI*Q0_mattawa_ (*n* = 1). Of the 14 mutations included in the Progenika panel, no cases of PI*S_iiyama_, PI*Q0_granite falls_ and PI*Q0_west_ were found. Other alleles not included in the initial 14-mutation panel were identified by gene sequencing. They included PI*M_würzburg_, PI*Z_bristol_, and PI*Z_wrexham_, and the null alleles PI*Q0_porto_, PI*Q0_madrid_, PI*Q0_brescia_, and PI*Q0_kayseri_.The frequency of rare and null alleles in the different countries are summarized in the Table 2.

**Table 1:** Rare and null alleles with their clinical characteristics by order of frequency

	n	Age (years)	Smoking habits			AAT available	AAT (mg/dl)	FEV_1_ available	FEV_1_ (%)
			Exsmoker	Never	Current				
All combinations	818	49.3 (19.8)	281 (34.4)	382 (46.7)	155 (18.9)	373 (45.6)	67.6 (28.0)	40.7 (49.8)	77.7 (27.3)
M/M _malton_	235	45.9 (20.8)	58 (24.7%)	127 (54.0%)	50 (21.3%)	110 (46.8)	78.2 (14.9)	88 (37.4)	80.7 (27.5)
M/I	126	54.8 (19.2)	51 (40.5%)	52 (41.3%)	23 (18.3%)	29 (23.0)	95.5 (28.4)	59 (46.8)	72.9 (25.4)
M/P _lowell_	105	47.2 (19.8)	35 (33.3%)	47 (44.8%)	23 (21.9%)	28 (26.7)	95.8 (28.2)	46 (43.8)	79.9 (20.1)
S/M _malton_	60	50.1 (21.1)	23 (38.3%)	26 (43.3%)	11 (18.3%)	110 (46.8)	48.1 (13.9)	41 (68.3)	80.2 (26.9)
S/I	36	48.1 (17.4)	16 (44.4%)	14 (38.9%)	6 (16.7%)	19 (52.8)	76.2 (24.1)	17 (47.2)	75.7 (30.0)
Z/M _malton_	30	51.2 (13.8)	14 (46.7%)	10 (33.3%)	6 (20.0%)	18 (60.0)	21.3 (14.8)	16 (53.3)	52.0 (30.9)
M/M _heerlen_	26	49.8 (20.6)	7 (26.9%)	15 (57.7%)	4 (15.4%)	14 (53.8)	71.5 (10.2)	16 (61.5)	77.9 (24.6)
M/F	23	52.9 (22.2)	8 (34.8%)	11 (47.8%)	4 (17.4%)	3 (13.0)	113.3 (14.9)	8 (34.8)	71.7 (30.0)
M/M _procida_	21	49.1 (19.5)	6 (28.6%)	7 (33.3%)	8 (38.1%)	12 (57.1)	72.5 (10.5)	16 (76.2)	81.7 (21.7)
M/Q0 _mattawa_	21	52.6 (19.8)	8 (38.1%)	11 (52.4%)	2 (9.5%)	11 (52.4)	63.4 (12.4)	13 (61.9)	83.2 (21.6)
S/P _lowell_	18	40.5 (16.4)	4 (22.2%)	10 (55.6%)	4 (22.2%)	14 (77.8)	77.1 (21.1)	11 (61.1)	96.1 (16.5)
M _malton_/M _malton_	13	52.2 (21.5)	5 (38.5%)	6 (46.2%)	2 (15.4%)	7 (53.8)	22.5 (6.8)	9 (69.2)	66.8 (28.9)
Z/P _lowell_	12	56.5 (18.9)	6 (50.0%)	4 (33.3%)	2 (16.7%)	9 (75.0)	38.9 (10.9)	9 (75.0)	90.7 (39.1)
Z/I	11	57.6 (13.4)	5 (45.5%)	3 (27.3%)	3 (27.3%)	8 (72.7)	64.0 (11.9)	7 (63.6)	76.8 (29.9)
M/Q0 _bellingham_	6	22.0 (17.4)	1 (16.7%)	5 (83.3%)	0 (0.0%)	6 (100)	78.2 (8.4)	3 (50.0)	92.3 (3.5)
F/Z	5	40.2 (30.9)	2 (40.0%)	2 (40.0%)	1 (20.0%)	4 (80.0)	76.0 (7.1)	5 (100)	58.0 (38.5)
F/S	4	45.2 (15.5)	1 (25.0%)	1 (25.0%)	2 (50.0%)	1 (25.0)	88.0	1 (25.0)	77.0
P _lowell_/P _lowell_	4	48.7 (9.2)	3 (75.0%)	1 (25.0%)	0 (0.0%)	3 (75.5)	57.0 (11.2)	3 (75.0)	71.6 (24.5)
S/M _heerlen_	4	42.5 (20.2)	2 (50.0%)	2 (50.0%)	0 (0.0%)	2 (50.0)	40.0 (0.0)	3 (75.0)	97.3 (6.4)
Z/M _procida_	4	64.0 (9.5)	0 (0.0%)	4 (100.0%)	0 (0.0%)	2 (50.0)	21.5 (0.7)	1 (25.0)	95.0
Z/Q0 _mattawa_	4	57.7 (12.7)	1 (25.0%)	2 (50.0%)	1 (25.0%)	3 (75.0)	23.6 (5.5)	2 (50.0)	73.5 (43.1)
S/M _procida_	3	63.3 (15.0)	2 (66.7%)	1 (33.3%)	0 (0.0%)	2 (66.7)	41.5 (4.9)	3 (100)	86.3 (29.2)
S/Q0 _mattawa_	3	55.6 (7.5)	1 (33.3%)	1 (33.3%)	1 (33.3%)	3 (100)	34.3 (11.9)	3 (100)	90.3 (20.3)
Z/M _heerlen_	3	58.6 (9.6)	2 (66.7%)	1 (33.3%)	0 (0.0%)	1 (33.3)	19.0	2 (66.7)	48.0 (26.8)
Z/M _palermo_	3	62.3 (6.6)	1 (33.3%)	2 (66.7%)	0 (0.0%)	1 (33.3)	20.0	1 (33.3)	31.0
Z/M _würzburg_	3	59.0 (15.6)	3 (100.0%)	0 (0.0%)	0 (0.0%)	3 (100)	47.6 (7.1)	3 (100)	92.0 (14.7)
I/P _lowell_	2	51.0 (14.1)	1 (50.0%)	1 (50.0%)	0 (0.0%)	2 (100)	65.5 (6.3)	2 (100)	107.0 (11.3)
M _malton_/M _heerlen_	2	41–0 (31.1)	1 (50.0%)	1 (50.0%)	0 (0.0%)	1 (50.0)	20.0	1 (50.0)	35.0
M/M _palermo_	2	41.5 (3.5)	0 (0.0%)	1 (50.0%)	1 (50.0%)	2 (100)	55.0 (1.4)	2 (100)	93.0 (18.3)
Z/Z _wrexham_	2	60.0 (1.4)	2 (100.0%)	0 (0.0%)	0 (0.0%)	1 (50.0)	57.0	1 (50.0)	29.0
M/Q0 _clayton_	1	72.0	0 (0.0%)	1 (100.0%)	0 (0.0%)	1 (100)	63.0	1 (100)	114.0
F/I	1	49.0	0 (0.0%)	1 (100.0%)	0 (0.0%)	0 (0.0)	–	0 (0.0)	–
F/Q0 _mattawa_	1	60.0	1 (100.0%)	0 (0.0%)	0 (0.0%)	1 (100)	65.0	1 (100)	27.0
I/M _heerlen_	1	88.0	0 (0.0%)	1 (100.0%)	0 (0.0%)	1 (100)	52.0	0 (0.0)	–
I/M _malton_	1	15.0	0 (0.0%)	1 (100.0%)	0 (0.0%)	0 (0.0)	–	0 (0.0)	–
M _heerlen_/M _heerlen_	1	49.0	1 (100.0%)	0 (0.0%)	0 (0.0%)	0 (0.0)	–	1 (100)	40.0
M _malton_/P _lowell_	1	51.0	1 (100.0%)	0 (0.0%)	0 (0.0%)	1 (100)	40.0	0 (0.0)	–
M _procida_/M _procida_	1	55.0	1 (100.0%)	0 (0.0%)	0 (0.0%)	0 (0.0)	–	0 (0.0)	–
M/M _malton_ + c.-428G > A + c.424C > T	1	54.0	1 (100.0%)	0 (0.0%)	0 (0.0%)	1 (100)	18.0	1 (100)	96.0
M/M _procida_ + c.194 T > C + c.853C > T	1	45.0	0 (0.0%)	1 (100.0%)	0 (0.0%)	1 (100)	20.0	0 (0.0)	–
M/M _procida_ + Q0 _porto_	1	55.0	1 (100.0%)	0 (0.0%)	0 (0.0%)	1 (100)	40.0	1 (100)	60.0
M/M _würzburg_	1	73.0	0 (0.0%)	1 (100.0%)	0 (0.0%)	1 (100)	3.0	1 (100)	70.0
M/P _lowell_ + c. − 109 + 41A > G	1	59.0	1 (100.0%)	0 (0.0%)	0 (0.0%)	1 (100)	52.0	1 (100)	75.0
M/P _lowell_ + Y _orzinuovi_	1	58.0	0 (0.0%)	1 (100.0%)	0 (0.0%)	1 (100)	54.0	1 (100)	135.0
M/Q0 _madrid_	1	61.0	1 (100.0%)	0 (0.0%)	0 (0.0%)	1 (100)	80.0	0 (0.0)	–
M/Q0 _mattawa_ + c.1052del	1	52.0	0 (0.0%)	1 (100.0%)	0 (0.0%)	1 (100)	5.0	1 (100)	31.0
M/Z _bristol_	1	69.0	0 (0.0%)	1 (100.0%)	0 (0.0%)	1 (100)	59.0	1 (100)	134.0
P _lowell_ / Z _bristol_	1	60.0	1 (100.0%)	0 (0.0%)	0 (0.0%)	1 (100)	22.0	0 (0.0)	–
P _lowell_/Y _orzinouvi_	1	37.0	1 (100.0%)	0 (0.0%)	0 (0.0%)	1 (100)	44.0	0 (0.0)	–
Q0 _brescia_/Q0 _brescia_ + c. − 10 T > C	1	55.0	1 (100.0%)	0 (0.0%)	0 (0.0%)	1 (100)	18.0	0 (0.0)	–
Q0 _kayseri_/Q0 _kayseri_	1	43.0	0 (0.0%)	1 (100.0%)	0 (0.0%)	1 (100)	29.0	0 (0.0)	–
Q0 _mattawa_/Q0 _mattawa_	1	68.0	0 (0.0%)	1 (100.0%)	0 (0.0%)	0 (0.0)	–	1 (100)	54.0
S/M _palermo_	1	77.0	0 (0.0%)	0 (0.0%)	1 (100.0%)	1 (100)	40.0	1 (100)	42.0
S/M _würzburg_	1	56.0	1 (100.0%)	0 (0.0%)	0 (0.0%)	1 (100)	40.0	1 (100)	53.0
S/Q0 _madrid_	1	56.0	0 (0.0%)	1 (100.0%)	0 (0.0%)	1 (100)	40.0	1 (100)	80.0
S/Z _bristol_	1	56.0	0 (0.0%)	1 (100.0%)	0 (0.0%)	1 (100)	56.0	1 (100)	131.0
S/Z _bristol_ + c.-428G > A + c.-10 T > C	1	74.0	0 (0.0%)	1 (100.0%)	0 (0.0%)	0 (0.0)	–	0 (0.0)	–

Data expressed as mean (standard deviation) or as absolute (relative) frequencies depending on the nature of the variable

*AAT* Alpha1 antitrypsin, *FEV1* Forced expiratory volume in 1 s

**Table 2:** Frequency of rare and null alleles in the different countries

	Argentina (*n* = 2,941)	Brazil (*n* = 2,620)	Chile (*n* = 3,352)	Colombia (*n* = 2,057)	LATAM (*n* = 10,520)	Spain (*n* = 18,272)	Turkey (*n* = 2,035)	All (*n* = 30,827)
Any mutation:	384 (15.4)	745 (28.4)	423 (12.6)	257 (12.5)	1809 (17.2)	7579 (41.5)	140 (6.9)	9528 (30.9)
Rare alleles	20 (0.8; 5.2)	66 (2.5; 8.9)	33 (1.0; 7.8)	6 (0.3; 2.3)	125 (1.2; 6.9)	576 (3.2; 7.6)	76 (3.7; 54.6)	777 (2.5; 8.2)
Null alleles	1 (0.0; 0.3)	6 (0.2; 0.8)	3 (0.1; 0.7)	0 (0.0; 0.0)	10 (0.1; 0.6)	31 (0.2; 0.4)	2 (0.1; 1.4)	43 (0.1; 0.4)
Rare + Null	21 (0.7; 5.4)	72 (2.7;9.6)	36 (1.0; 8.5)	6 (0.2; 2.3)	135 (1.2; 7.4)	607 (5.7; 8.0)	78 (3.8; 55.7)	820 (2.7; 8.6)

Data expressed as absolute numbers with percentages in parenthesis; first value showing percentages referred to the total number of samples in the geographical area, second value showing percentages referred to the total number of cases with mutations in the geographical area

**Fig. 1:**
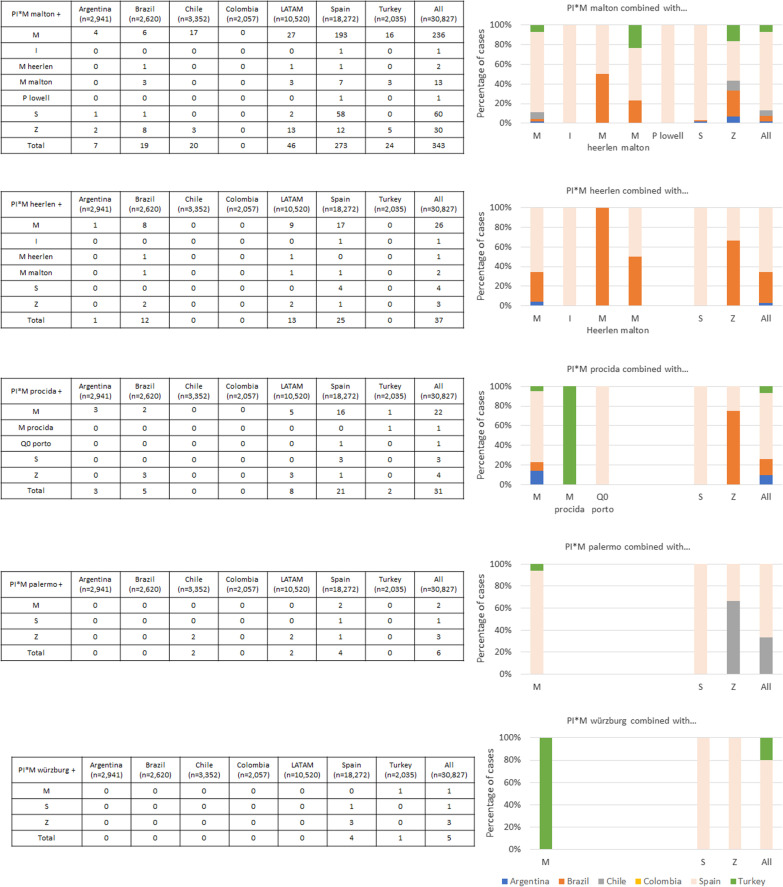
M-like alleles distribution

The frequency of the different M-like rare alleles is shown in Fig. 1. The most frequent M-like allele was PI*M_malton_ followed by PI*M_heerlen_. Although these alleles were identified predominantly in the samples from Spain, some combinations (PI*M_malton_, PI*M_heerlen_ or PI*M_procida_) were found in samples from other countries. After Spain, Brazil had the most of these rare mutations.

**Fig. 2:**
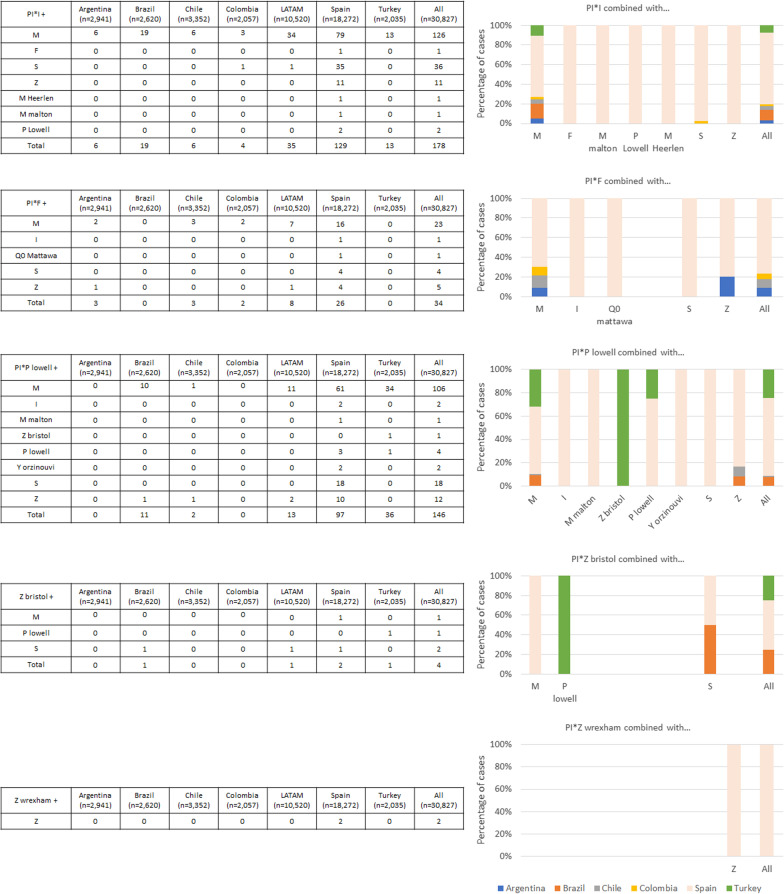
Other rare alleles distribution

The frequency of other rare alleles is shown in Fig. 2. PI*I was the most common and was predominantly found in samples from Spain. The allele PI*F was also frequently identified. Other alleles were less frequent, but some were identified in Turkey, e.g., combinations with P_lowell_.

**Fig. 3:**
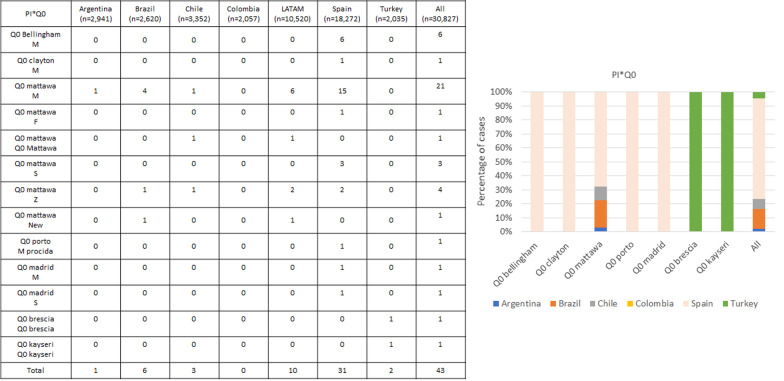
Null alleles distribution

The null alleles are summarized in Fig. 3. The most frequent null allele was Q0_mattawa_. These alleles were less frequent, and homozygous combinations were extremely rare. The Q0_kayseri_ mutation is native to Turkey, but the only homozygous case for Q0_brescia_ was also found in a sample from that country. Information on these mutations from a non-systematic literature review is summarized in Table 3.

**Table 3:** Summary of the main characteristics of the mutations found

Allele name	Base	Nucleotide change	Amino acid change	SNP code	ClinVar code	AAT level (%)	AAT activity	Lung risk	Liver risk	References
F		c.739C > T	p.Arg247Cys	rs28929470	17,961	80	Reduced	Low	Low	[22]
I		c.187C > T	p.Arg63Cys	rs28931570	17,974	50	Reduced	Low	Low	[22]
M_procida_		c.194 T > C	p.Leu65Pro	rs28931569	17,971	10	Slightly reduced	High	Low	[40]
M_heerlen_		c.1178C > T	p.Pro393Leu	rs199422209	17,965	2	Reduced	High	Low	[38]
M_wurzburg_	M1V	c.1177C > T	p.Pro393Ser	rs61761869	289,135	10–15	Reduced	High	Low	[36]
M_vall d’hebron_	M1A							High	Low	[37]
M_palermo_	M1V	c.227_229delTCT	p.Phe76del	rs775982338	315,028	15	Reduced	High	High	[33]
M_malton_	M2					15	Reduced	High	High	[31]
M_nichinan_†		c.227_229delTCT	p.Phe76del	rs775982338	315,028	15	Reduced	High	High	[32]
		c.514G > A	p.Gly172Arg	rs112030253	393,473					
P_lowell_	M1V	c.839A > T	p.Asp280Val	rs121912714	17,975	40	Reduced	High	High	[42]
P_duarte_ Q0_cardiff_	M4					40	Reduced	High	Low	[43]
Q0_bellingham_		c.721A > T	p.Lys241*	rs199422211	17,977	Undetectable		High	Low	[52]
Q0_granitefalls_		c.552delC	p.Tyr184*	rs267606950	17,976	Undetectable		High	Low	[53]
Q0_madrid_	M3	c.-5 + 2dupT	NA	Not Reported	Not Reported	Undetectable		High	Low	[54]
Q0_faro_	M1V					Undetectable		High	Low	
Q0_mattawa_	M1	c.1130dupT	p.Leu377Phefs*24	rs763023697	552,891	Undetectable		High	Low	[55]
Q0_ouren_	M3					Undetectable		High	Low	[56]
Q0_west_		c.646 + 1G > T	NA	rs751235320	189,064	Undetectable		High	Low	[57]
Q0_clayton_ Q0_saarbruecken_		c.1158dupC	p.Glu387Argfs*14	rs764325655	188,845	Undetectable		High	Low	[58] [33]
S_iiyama_		c.230C > T	p.Ser77Phe	rs55819880	17,992	30	Slightly reduced	High	High	[59]
Y_barcelona_‡		c.839A > T	p.Asp280Val	rs121912714	17,975	10	Reduced	High	High	[21]
		c.1244C > A	p.Pro415His	Not Reported	Not Reported					
Y_orzinuovi_		c.1244C > A	p.Pro415His	Not Reported	Not Reported	10	Reduced	High	High	[46]
Z_bristol_	M1V	c.326C > T	p.Thr85Met	rs199422213	17,993	15	Reduced	High	High	[48]
Z_wrexham_		c.17C > T	p.Ser6Leu	rs140814100	17,970	15	Reduced	High	High	[51]

RefSeq: NM_001127701.1

^†^The M_nichinan_ variant has been described as a haplotype that combines the M_malton_ and c.514G > A variants (a variant that does not seem to have a deleterious effect on its own)

^‡^Y_barcelona_ results from the combination of P_lowell_ and Y_orzinuovi_

*NA* not applicable

## Discussion

The present study assessed the frequency of rare mutations in a large sample of cases with suspected AATD in six countries. Our results show the low frequency of these alleles and their distribution in different countries and help identify which variants are more frequent in different geographical areas. Our data indicate that these so-called rare variants may not be as rare when a thorough diagnostic system is used.

AATD is an inherited disorder that increases the risk of lung and liver disease. Numerous point mutations of the *SERPINA1* gene have been identified so far, although many of them are not associated with an increased risk for developing respiratory or liver disorders [2]. Consequently, the identification of less frequent, but consequential mutations and their characterization are relevant objectives for the management of AATD. Greater understanding of the underlying biologic pathways leading to cell damage in AATD will also be of benefit for the treatment of AATD [7]. This is of special importance in the current pandemic situation with potential associations between AATD and COVID19 [8, 9]. The Progenika diagnostic network is formed by those countries using the Progenika system as the diagnostic standard for AATD. Other countries have started to use a similar system including Italy [10] and Germany [11].

The main strengths of our study are the large number of samples analyzed, the simultaneous determination of several genotypes and the sequencing of samples from different countries, allowing the assessment of the geographic distribution of these mutations. However, there are some limitations that must be taken into account when interpreting our results. This is not a population-based study, but a highly selected population of patients with suspected AATD. Accordingly, the prevalence figures may overestimate the prevalence of AATD in the general population. Another note of caution should be considered in the cases with hepatopathy of unknown cause. The clinicians participating in this circuit were mostly pulmonologists or general practitioners. Therefore, cases with hepatopathy of unknown cause may be under-represented. The addition of liver disease specialists to the evaluation of these patients might contribute to the detection of cases of AATD in this clinical context. Additionally, not all samples were sequenced, only those with a discrepancy between the serum level of AAT and the mutation found. There was a considerable number of cases with no AAT level available. Therefore, there may be an underestimation of some alleles. Finally, serum AAT and FEV_1_ reported in Table 1 are influenced by the other accompanying allele in heterozygosis. Consequently, these data may lead to a false picture of the impact of these alleles on AAT levels or the resulting functional impairment. Interestingly, the majority of cases with AAT values available presented as non-severe AATD, suggesting that these alleles cannot be ruled out by the level of serum AAT alone.

Despite these limitations, this is the largest study to date that includes analysis of the frequency of rare variants in a sample of patients with suspected AATD. The frequency of these rare alleles has been previously reported in several individual countries including Germany [12], Italy [13, 14], Tunisia [15], Switzerland [16], Spain [17], Poland [18], Turkey [19] and the USA [20]. In these studies, the frequency of rare alleles ranged from 0.5% of all screened patients in Germany [12] to 4.1% in Tunisia [15] corresponding to 1.7% of cases with any mutation in Germany [12] and 20% in Tunisia [15]. In Turkey, our data showed a higher frequency of rare alleles, in line with recently published data from this country [19] within the Progenika network.

The information obtained from our literature review should be interpreted with caution since some mutations have low case numbers, and their effects may be influenced by an accompanying mutation. Additionally, some mutations have been assigned more than one name. There were two major allele complexes that are worth noting. The M_malton_ complex includes the M_malton_ (c.227_229delTCT on M2 variant), M_palermo_ (same mutation on M1V variant) and M_nichinan_ (same mutation with an additional mutation c.514G > A that does not seem to have a deleterious effect on its own). The P_lowell_ complex includes P_lowell_ (c.839A > T on M3 variant) and P_duarte_, (same mutation on M1 variant; also known as Q0_cardiff_). The P_lowell_ mutation is also seen in Y_barcelona_ which results from the combination of P_lowell_ and Y_orzinuovi_ in the same gene [21].

PI*I and PI*F were first alleles described in 1967 [22]. PI*I allele has been associated with moderate AATD with serum concentrations similar to those observed with the S allele [23]. PI*II homozygotes usually have AAT levels around 50 mgr/dL [24, 25]. Liver involvement is not usually seen with PI*I unless it is accompanied by an allele associated with liver involvement [26]. The serum concentration and function associated with the PI*F allele are at least 80% of that of the M allele [27, 28]. However, the PI*F allele shows a decreased ability to bind and less time-dependent inhibition of human neutrophil elastase compared to the M phenotype and similar inhibition to that of the Z phenotype [29]. The PI*F allele has a reduced functional ability to inhibit neutrophil elastase but not proteinase 3 [30], suggesting that inheritance of the F variant may increase a person's susceptibility to elastase-induced lung damage, but not necessarily to emphysema. Due to normal hepatic secretion, it does not produce intrahepatic accumulation and therefore, does not increase the risk of liver injury.

According to our results, M-like alleles are the most frequent in patients with suspected AATD. PI*M_malton_ complex (PI*M_malton_, PI*M_palermo_ and PI*M_nichinan_) have a similar behavior. PI*M_malton_ was first described in 1975 in a 2-year child with a minor infection [31]. PI*M_nichinan_ was first described in 1990 in a Japanese individual with severe AATD (18 mg/dl), associated with aggregated AAT molecules in the hepatocytes [32]. Finally, PI*M_palermo_ was first described in 1994 [33]. Their presence is associated with serum AAT levels below 15%. These mutations are characterized by conformational abnormalities that result in polymerized/aggregated insoluble forms of AAT that accumulate in the endoplasmic reticulum of hepatocytes. Therefore, all three variants meet the requirements for endoplasmic reticulum storage diseases and conformational diseases [34, 35]. Interestingly, the c.514G > A additional mutation of the PI*M_nichinan_ does not contribute to AATD [32].

PI*M_würzburg_ was first described in 1999 on a M1Val basis [36], and the same mutation was identified one year after as PI*M_vall d’hebron_ but on a M1Ala basis [37]. These defective alleles produce a change in the amino acid sequence at position 369 which is associated with a complete intracellular transport block in cell. Interestingly, the allele PI*M_heerlen_ has a different amino acid substitution in the same position which is also shown to cause complete retention of the mutant protein in the hepatocytes.

PI*M_heerlen_ was first described in 1981 [38]. Homozygous cases have serum AAT levels 2% of normal and very low antitrypsin activity. The tertiary structure of the M_heerlen_ protein is significantly altered resulting in intracellular proteolysis. Therefore, there is no accumulation of M_heerlen_ protein in hepatocytes [39].

PI*M_procida_ was first described in 1988 [40]. This rare allele encoding AAT synthesis is associated with reduced serum AAT levels (below 10 mg/dl). The M_procida_ molecule behaves normally in vivo with a half-life similar to normal M1 AAT. Neutrophil elastase inhibitory activity of M_procida_ protein is slightly reduced. Evaluation of the crystallographic structure suggests that the mutation may alter alpha-helix A, suggesting that the molecule is unstable and degrades intracellularly prior to secretion. The tertiary structure of the protein is significantly altered resulting in intracellular proteolysis and, therefore, not associated with risk of liver injury. The risk of lung disease is high, but the risk of liver disease is low [40].

Although P-type mutations have been known since 1968 [41], it was not until 1990 that the PI*P_lowell_ genotype began to be characterized [42]. In 1993, a new P-allele was identified as P_duarte_ which carried the same mutation as P_lowell_ but on a M4 basis [43]. These alleles have similar behavior. Homozygous P_lowell_ exhibits decreased AAT serum concentration—around 40% of normality [44]. However, P_lowell_ has near normal function as an inhibitor of human neutrophil elastase [45]. Therefore, increased risk for lung involvement depends on the accompanying alleles [41]. The P_lowell_ substitution has a profound effect on intracellular processing of the AAT molecule resulting in deficiency. This variant has been associated with increased intracellular degradation of newly synthetized protein and to serum levels 24% of normal [42]. Therefore, the risk for liver disease is low. PI*P_duarte_ is similar to P_lowell_ but on M4. AAT levels in P_duarte_ are 41% of normal, similar to P_lowell_ [43]. Thus, the P_duarte_ allele differs from the P_lowell_ allele only by the normal allelic background in which the mutation occurs.

Y_barcelona_ was first described in 1998 as the combination of PI*P_lowell_ + another mutation (c.1244C > A) [21]. In 2012, the mutation c.1244C > A was reported to have a pathogenetic effect by itself, i.e., a case with mild hyper-transaminasemia reported in Orzinuovi (Brescia, Italy). The allele was named as PI*Y_orzinuovi_ [46]. Consequently, Y_barcelona_ results from a combination of PI*P_lowell_ plus PI*Y_orzinuovi_. In heterozygous cases, the risk of lung disease is likely to be similar to that of MZ heterozygotes [47].

Z_bristol_ was first reported in 1997 in a woman with an obstetric history of three perinatal deaths from fulminant liver disease and no living offspring [48]. Only a few cases have been reported in children with low levels of AAT if accompanied by a Z allele and near to normal if accompanied by an M allele, with frequent liver involvement in children [49, 50]. The Z_wrexhan_ allele has only been described in a family with severe AATD which also carried the common mutation causing Z deficiency [51]. Individuals with such a deficiency are, therefore, compound heterozygotes. The behavior of these particular mutations in the absence of the Z mutation is not known.

Null (Q0) alleles encode a truncated protein with large conformational changes that is degraded intracellularly without having the opportunity to aggregate. These patients have undetectable serum concentrations of AAT. The protein is retained in the rough endoplasmic reticulum or pre-Golgi compartment and is degraded. This means that homozygotes are at very high risk for emphysema, but not liver disease.

In conclusion, the present report informs on the frequency of rare and null alleles updating their distribution in a large sample population from six countries. The Progenika diagnostic network has allowed the identification of several rare alleles providing a new view of the distribution of these alleles in different countries. Due to the efficacy in both the detection of AATD cases and the identification of new variants, in the future we believe that Progenika's system could continue to expand to other countries. Consequently, future studies should focus on the characterization of these and other new mutations as they emerge in the context of patients with suspected AATD. These findings may help prioritize allele selection for routine testing and highlights the need for continuing research into their pathogenetic roles.

## Data Availability

All data generated or analyzed during this study are included in this published article and its supplementary information files.

## Abbreviations

AATD: Alpha1 antitrypsin deficiency
FEV_1_: Forced expiratory volume in one second
